# “Funducation”—The New Age of Learning, Intersection of Education, and Fun

**DOI:** 10.1055/a-2351-9736

**Published:** 2024-07-18

**Authors:** Joon Pio Hong, Jaume Masià

**Affiliations:** 1 Department of Plastic and Reconstructive Surgery, Asan Medical Center, University of Ulsan College of Medicine, Seoul, Republic of Korea; 2 Department of Plastic and Reconstructive Surgery, Hospital de la Santa Creu i Sant Pau, Barcelona, Spain

**Figure:**
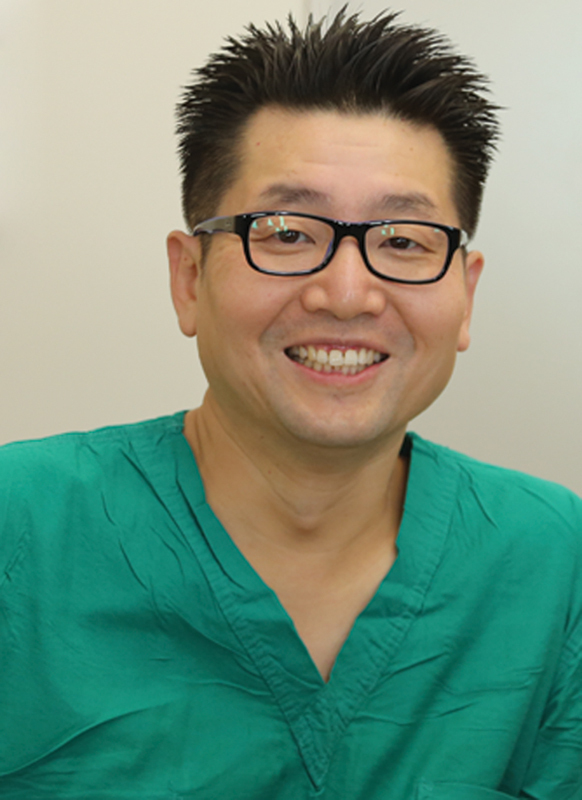
Joon Pio Hong, MD, PhD, MMM, (Editor-in-Chief)

**Figure:**
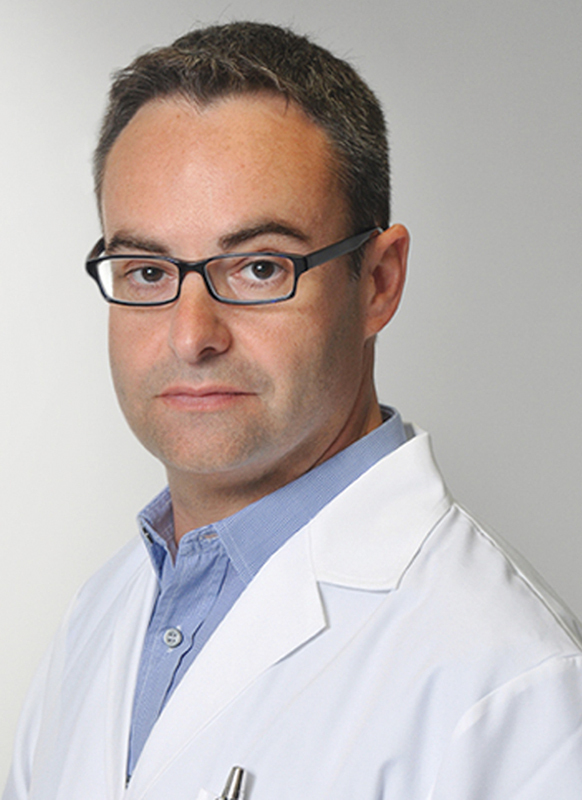
Jaume Masià, MD, PhD, (Associate Editor)

Can education be fun? Education is a serious and important part of life that helps people grow professionally. This can be even more so for medical professionals where mistakes can lead to critical outcomes. Treating patients may be far from fun and often grave but first, we need to have the right knowledge to provide the service hence the master–apprentice model has been used for a long time. However, modern medicine is a field of continuous evaluation and change but often it is difficult to accommodate change when our minds are fixed on finding solutions from what we learned. Then how can we educate the next generation of physicians or health care workers to have an innovative mindset while maximizing the learning experience?

Already at 350 BC, Socrates encouraged students to ask questions, think critically, and come up with their answers or hypotheses. This is the essence of interaction that we speak of today. When students or participants are engaged, they feel a sense of participation, motivation, and achievement. The young generation today is known for a very short attention span but who would have a long span if meeting or the educational process is rigid and too serious?

The recent meeting at the Barcelona Breast Meeting was a very good example of how fun and education can be mixed to bring out the best possible learning experience. Bring new technology and participants were able to try the new platform at the meeting, boxing session where debates were thrown at each other like a boxing match, the cooking session where master surgeons dressed like chefs shared their secret recipe for successful outcomes, cinema paradiso where a surgery video presentation was like a movie drama, and all these sessions were so engaging and most of all fun to watch and easy to participate. This may be the future of education to enhance participation through the element of fun, we like to call it Fun and Education, “Funducation.”

Overall, incorporating elements of fun into education can help to make learning more enjoyable and effective. By engaging students through games, humor, creativity, and hands-on experiences, educators can create a more dynamic and engaging learning environment that fosters curiosity and a love of learning. Most of all helping them to understand the logic and to help them build their logic is the key to innovation. Meetings where asking questions and debating through a fun process can be less intimidating and more engaging. Now it is the proper time for scientific meetings to evolve exploring the Socratic approach with the element of fun.

